# Could glucose be a proaging factor?

**DOI:** 10.1111/j.1582-4934.2008.00329.x

**Published:** 2008-04-08

**Authors:** Eva Kassi, Athanasios G Papavassiliou

**Affiliations:** Department of Biological Chemistry, Medical School, University of AthensAthens, Greece

**Keywords:** AGEs, aging, autophagy, glucose, glycolysis, mTOR signalling, oxidative stress, sirtuins

## Abstract

There is an ever-increasing scientific interest for the interplay between cell's environment and the aging process. Although it is known that calorie restriction affects longevity, the exact molecular mechanisms through which nutrients influence various cell signalling/modulators of lifespan remain a largely unresolved issue. Among nutrients, glucose constitutes an evolutionarily stable, precious metabolic fuel, which is catabolized through glycolytic pathway providing energy in the form of ATP and consuming NAD. Accumulating evidence shows that among the important regulators of aging process are autophagy, sirtuin activity and oxidative stress. In light of recent work indicating that glucose availability decreases lifespan whilst impaired glucose metabolism extends life expectancy, the present article deals with the potential role of glucose in the aging process by regulating – directly through its metabolism or indirectly through insulin secretion – autophagy, sirtuins as well as other modulators of aging like oxidative stress and advanced glycation end-products (AGEs).

## Introduction

Aging is a complex, multi-factorial process and numerous aging theories have been proposed. Evolutionarily conserved genes and pathways have been shown to regulate lifespan in mammals. Many gene products known to affect lifespan are intimately involved in the control of energy metabolism including the fuel sensor AMP-activated protein kinase (AMPK) [1], while the lack of *klotho* gene expression – an important gene for the maintenance of normal energy homeostasis – is characterized by various systemic phenotypes resembling human aging [2].

Although it is clear that our genes influence aging and longevity, how genes interplay with environmental factors (*i.e.*nutrition, exercise, stress) and how exactly this takes place on a molecular level is still only partially understood and remains a big challenge for scientists.

Glucose, the major form in which carbohydrates absorbed from the intestinal tract is presented to the cells constitutes a very important energy source for the body, serving for some tissues as a vital metabolic fuel; it's catabolic pathway, glycolysis, represents an ancient process employed by all body cells to extract part of the chemical energy inherent in the glucose molecule.

In view of current data supporting that glucose availability decreases *Caenorhabditis elegans* lifespan whereas impaired glucose metabolism extends life expectancy [3], a question arises: could this precious fuel, glucose and its catabolic pathway, glycolysis, be implicated in the aging process?

Here, we briefly discuss how glucose could directly (through its metabolism) or indirectly (by provoking insulin secretion) affect two of the main regulators of the aging process, autophagic and sirtuin activity, as well as other factors involved in aging such as oxidative stress and advanced glycation end-products (AGEs).

## Autophagy

Autophagy is a process of degradation and recycling of most longlived proteins, biological membranes, macromolecules and entire organelles like mitochondria, ribosomes etc., thus playing a crucial role in homeostasis of living cells [4]. Autophagy occurs constitutively at low levels even under normal growth conditions and it seems that baseline autophagy is critical for intracellular clearance.

Various human pathologies are associated with decreased autophagic activity, especially in non-dividing cells of the nervous and muscle systems where turnover of long-lived intracellular proteins and organelles may be very important. On the other hand, augmented autophagic activity in aging organisms can have preventive potential [5]. Calorie restriction-induced autophagy has a well-documented effect in extending life expectancy in mammals [6].

**Fig. 1:**
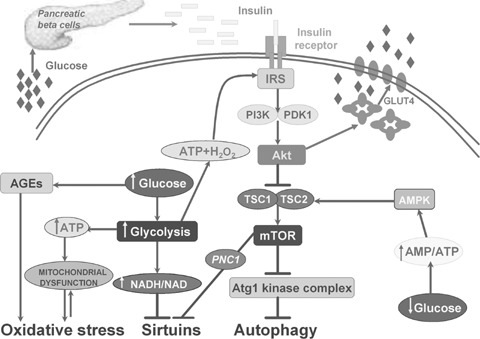
Molecular mechanisms of autophagy, sirtuins and oxidative stress regulation by glucose. Glucose can directly (through its metabolism) or indirectly (by provoking insulin secretion from pancreatic beta cells) affect the main regulators of the aging process, autophagic and sirtuin activity as well as other contributors to aging like oxidative stress and advanced glycation end-products (AGEs). Insulin receptor activation leads to a phosphatidylinositol-3 kinase (PI3K)- / phosphoinositide-dependent protein kinase 1 (PDK1)- / serine-threonine kinase PKB (Akt)- and mammalian target of rapamycin (mTOR)-mediated suppression of autophagy. In the presence of adenosine-5′-triphosphate (ATP) and hydrogen peroxide, which both can be produced by increased glycolysis, the insulin receptor can be activated even in the absence of insulin. Under ATP privation through decreased intracellular glucose offer, the AMP-activated protein kinase (AMPK) phosphorylates and potentiates tuberous sclerosis protein 2 (TSC2) which inhibits mTOR in combination with TSC1 (hamartin). An increased glycolytic activity would tend to provoke an accumulation of NADH and lower NAD availability, resulting in decreased sirtuin activity. mTOR can also suppress sirtuin activity through inhibition of *nicotinamidase* gene *(PNC1)* expression. Finally, an increased intracellular glucose offer can lead *via* increased glycolysis to: (*i*) mitochondrial dysfunction and oxidative stress (increased reactive oxygen species) due to continuous ATP synthesis, and (*ii*) accumulation of highly toxic advanced glycation end-products (AGEs) which can further provoke oxidative stress. GLUT4, glucose transporter 4; IRS, insulin receptor substrate.

It is well known that extracellular glucose levels are connected to cell metabolism, at least in part, through insulin signalling. Insulin, whose secretion is triggered by glucose levels, is thought to be one of the major suppressive factors for autophagy. Interestingly, decreased insulin signalling is linked to enhanced longevity in worms, flies and mice [7], posing that insulin resistance might be a defence mechanism against aging. Insulin receptor activation leads to the phosphorylation of key tyrosine residues on insulin receptor substrate(IRS) proteins, resulting, in turn, in a phosphatidylinositol-3 kinase (PI3K)-/ phosphoinositide-dependent protein kinase 1 (PDK1)- / serine-threonine kinase PKB (Akt)-and mammalian target of rapamycin (mTOR)-mediated suppression of autophagy [8] (Fig. 1). The rate of phosphorylation of the insulin receptor kinase domain and several downstream targets including the phosphatidylinositol phosphates, Akt1 and mTOR is determined by the balance between kinase and phosphatase activities. Notably, in the presence of adenosine-5′-triphosphate (ATP) and hydrogen peroxide, which both can be produced by increased glucose intake [9], the insulin receptor kinase domain is phosphorylated at its catalytic site and thereby rendered catalytically active even in the absence of insulin [10]Fig. 1. In contrast, under ATP privation, the AMPK phosphorylates and potentiates tuberous sclerosis protein 2 (TSC2), which inhibits mTOR in combination with TSC1 (hamartin) [11] (Fig. 1). Interestinlgy, an over-expression of an AMPK alpha subunit (aak-2) in *Caenorhabditis elegans* has been shown to increase lifespan [1].

Thus, glucose could activate insulin receptor signalling pathway either *via* induction of insulin secretion by β cells or even in the absence of insulin through increased ATP and hydrogen peroxide production, hence leading to an activated mTOR complex which, by acting on the Atg1 kinase complex, inhibits several steps in autophagosome formation [12]. To this end, mTOR has been shown to affect negatively lifespan [13].

## Sirtuins

Among the genes that have been reported to regulate aging in different species are *SIR2* and its functional orthologs, sirtuins. Sirtuin proteins are a family of protein deacetylases, which control a diverse array of pathways involved in the aging process [14]. They can forestall aging by stabilizing the *rDNA* locus [15] and recently have been found to promote mitochondrial biogenesis in liver and muscle through the transcriptional coactivator peroxisome proliferator-activated receptor γ coactivator-1α (PGC-1a) [16, 17], and cell survival by deactivating the tumour suppressor p53 [18].

Sirtuins are unique in that they require nicotinamide adenine dinucleotide (NAD) as a cofactor [19, 20]. In a complicated reaction, sirtuins couple lysine deacetylation to NAD hydrolysis, yielding *O*-acetyl-ADP-ribose and nicotinamide [20]. As such, sirtuin activity seems to be governed by cellular [NAD]/[NADH] ratios and respond to changes in cellular metabolism [21, 22]. Indeed, recent studies imply that the metabolism of NAD/NADH controls cell senescence and lifespan *via* regulating sirtuin activity [23, 24].

As mentioned above, glycolysis is used by all body cells to obtain part of the chemical energy entrapped in the glucose molecule. Glycolytic pathway is known to lead to the net production of ATP, consuming NAD during the metabolism of glyceraldehyde-3-phosphate to 1, 3-diphosphoglycerate and NADH (as products). An increased glycolytic activity would tend to provoke an accumulation of NADH and lower NAD availability, resulting in a decreased sirtuin activity (Fig. 1).

Nonetheless, this is not the only mode of interplay between glucose and sirtuin activity. A recent study suggested that mTOR inhibition promotes longevity by re-localizing two transcription factors, Msn2p and Msn4p, from the cytoplasm to the nucleus, whereby they stimulate expression of the nicotinamidase gene *PNC1*, a modulator of sirtuin activity [25] (Fig. 1). Consequently, a glucose challenge, by potentiation of insulin/insulin receptor signalling cascade could down-regulate sirtuin activity through mTOR signalling.

In conclusion, an insulin-dependent or independent high-intra-cellular glucose offer in combination with a hyperactivation of glycolytic pathway could down-regulate autophagy (through insulin receptor signalling cascade) and sirtuin activity (either *via* insulin receptor signalling cascade or through decreased NAD levels) at the same time.

## Oxidative stress and AGEs

A progressive rise of oxidative stress and related inflammatory reaction appears to play a crucial role in the aging process and many age-related diseases. Glucose and glycolysis have also been implicated in the induction of oxidative stress.

Indeed, in an increased intracellular glucose offer, due to continuous ATP synthesis *via* increased glycolysis, less ATP is required from mitochondrial function, so that the decreased supply of electrons (as acetyl-CoA or from NADH) to the electron transport chain would tend to produce more incompletely reduced oxygen species, that is oxygen-free radicals. Any increased intramitochondrial reactive oxygen species (ROS) production could also increase the probability of mitochondrial dysfunction leading to a vicious cycle [26] (Fig. 1). Schulz *et al.*[3] found that glucose deprivation in *Caenorhabditis elegans* induced a mild oxidative stress – by ROS formation –, which could promote the development of antioxidant defence mechanisms that protect the organism from oxidative molecular damage associated with aging. On the other hand, recent studies have shown that glucose challenge induced a consistent increase in ROS generation by human polymorphonuclear and mononuclear leucocytes [9], an effect that was not produced by equicaloric amounts of alcohol [27]. Moreover, glucose challenge increased intracellular NF-κB, a master regulator of inflammation-aging process, which normally is inhibited by sirtuins [28]. Reinforcing the above, recent work revealed that in response to oxidative stress, organisms can re-direct their metabolic flux from glycolysis to the pentose phosphate pathway by regulating key glycolytic enzymes like triosephosphate isomerase and glyceraldehyde-3-phosphate dehydrogenase (GAPDH), and this ability is conserved between yeast and animals indicating its importance in the adaptation to oxidative stress [29].

An hyperactivation of glycolytic pathway could also lead to accumulation of glyceraldehyde-3-phosphate and dihydroxyacetone phosphate (DHAP), which both can glycate proteins and putrefied to methylglyoxal, a highly toxic and very reactive glycating agent [30]. It is known that protein AGEs are increased under conditions of hyperglycaemia [31]. Protein AGEs can themselves induce inflammatory conditions and provoke production of ROS, which can further influence cell function (Fig. 1). Specifically, methylglyoxal has been shown to trigger many of the deleterious physiological and biochemical changes characteristic of the aged phenotype, including increased ROS generation, mitochondrial dysfunction, apoptosis and inhibition of cell division [32].

According to recent reports, increased dietary AGE intake accelerates aging and decreases lifespan, whereas decreasing dietary AGE intake can preserve defence functions against oxidative stress, decrease tissue damage in humans and extend lifespan in mice [33–34]. It is therefore conceivable that decreasing metabolically generated protein AGEs could help lowering the overall AGE load and could have beneficial effects by suppressing aging and extending lifespan.

Moreover, when the sticky ends of AGEs adhere to neighbouring proteins, they form permanent, disabling cross-links. Proteases, which normally broke down damaged proteins, are inhibited in the presence of cross-linkage, thus leading to accumulation of damaged protein molecules. Indeed, atherosclerosis, cataract, skin alterations, renal dysfunction are some of the age-related conditions that are partially attributed to glycation processes.

## Considerations

Conceivably, factors affecting the intracellular glucose offer (*i.e.* increased expression of glucose transporters; augmented activation of insulin signalling by increased secretion of insulin due to glucose challenge or by post-receptor signals) as well as the activity of glycolytic pathway (*i.e.* hyperactivation due to defects in glycolytic enzymes-regulators of positive/ negative feedback mechanisms, which could lead to low levels of NAD and increased ATP generation hence lower mitochondrial function – with consequently impaired regeneration of NAD by NADH – and increased intramitochondrial ROS production), could account for an increased ‘proaging’ activity. It should be noted that an increase in lifespan can be achieved in yeast by a reduction of glucose in the growth media or by mutations that reduce the metabolism of glucose (such as deletion of *hexokinase 2)*[35], whereas in klotho mouse – which genetically lacks *klotho* gene expression, encoding a β-glucosidase-like protein – an augmented expression of glucose transporter 4 (GLUT4) and an increased insulin sensitivity have been demonstrated [36].

## Conclusions

Since until very recently the organisms' main problem was dealing with nutrient deprivation (rather than the current surfeit); it was more economical during times of caloric restriction to shut off reproduction, decrease hormonal activity and decrease metabolic activity until nutrients again became available. It was evolutionarily more advantageous to keep the organism alive longer when it was starving than to expend energy reproducing and thus create more competition for limited nutrients.

Indeed, since the 1930s it is known that limiting the food consumed by laboratory rodents increases their lifespan [37]. Eighty years later, having our knowledge in the interaction between nutrients and life expectancy widen, we may say that one of this nutrients, glucose, could be a pro-aging factor by affecting important regulators of the aging process, while restriction of glucose could trigger physiological changes linked to health and longevity.

## References

[1] Curtis R, O'Connor G, DiStefano PS (2006). Aging networks in Caenorhabditis elegans: AMP-activated protein kinase (aak-2) links multiple aging and metabolism pathways. Aging Cell.

[2] Mori K, Yahata K, Mukoyama M, Suganami T, Makino H, Nagae T, Masuzaki H, Ogawa Y, Sugawara A, Nabeshima Y, Nakao K (2000). Disruption of klotho gene causes an abnormal energy homeostasis in mice. Biochem Biophys Res Commun.

[3] Schulz TJ, Zarse K, Voigt A, Urban N, Birringer M, Ristow M (2007). Glucose restriction extends *Caenorhabditis elegans* lifes-pan by inducing mitochondrial respiration and increasing oxidative stress. Cell Metab.

[4] Wang CW, Klionsky DJ (2002). The molecular mechanism of autophagy. Mol Med.

[5] Terman A, Gustafsson B, Brunk UT (2007). Autophagy, organelles and ageing. J Pathol.

[6] Del Roso A, Vittorini S, Cavallini G, Donati A, Gori Z, Masini M, Pollera M, Bergamini E (2003). Ageing-related changes in the *in vivo* function of rat liver macroautophagy and proteolysis. Exp Gerontol.

[7] Barbieri M, Bonafè M, Franceschi C, Paolisso G (2003). Insulin/IGF-I-signaling pathway: an evolutionarily conserved mechanism of longevity from yeast to humans. Am J Physiol Endocrinol Metab.

[8] Mizushima N (2005). The pleiotropic role of autophagy: from protein metabolism to bactericide. Cell Death Differ.

[9] Mohanty P, Hamouda W, Garg R, Aljada A, Ghanim H, Dandona P (2000). Glucose challenge stimulates reactive oxygen species (ROS) generation by leucocytes. J Clin Endocrinol Metab.

[10] Dröge W, Schipper HM (2007). Oxidative stress and aberrant signaling in aging and cognitive decline. Aging Cell.

[11] Inoki K, Zhu T, Guan KL (2003). TSC2 mediates cellular energy response to control cell growth and survival. Cell.

[12] Kamada Y, Funakoshi T, Shintani T, Nagano K, Ohsumi M, Ohsumi Y (2000). Tormediated induction of autophagy *via* an Apg1 protein kinase complex. J Cell Biol.

[13] Kapahi P, Zid BM, Harper T, Koslover D, Sapin V, Benzer S (2004). Regulation of lifespan in Drosophila by modulation of genes in the TOR signaling pathway. Curr Biol.

[14] Hekimi S, Guarente L (2003). Genetics and the specificity of the aging process. Science.

[15] Fritze CE, Verschueren K, Strich R, Easton Esposito R (1997). Direct evidence for SIR2 modulation of chromatin structure in yeast rDNA. EMBO J.

[16] Rodgers JT, Lerin C, Haas W, Gygi SP, Spiegelman BM, Puigserver P (2005). Nutrient control of glucose homeostasis through a complex of PGC-1alpha and SIRT1. Nature.

[17] Lerin C, Rodgers JT, Kalume DE, Kim SH, Pandey A, Puigserver P (2006). GCN5 acetyltransferase complex controls glucose metabolism through transcriptional repression of PGC-1alpha. Cell Metab.

[18] Cheng HL, Mostoslavsky R, Saito S, Manis JP, Gu Y, Patel P, Bronson R, Appella E, Alt FW, Chua KF (2003). Developmental defects and p53 hyper-acetylation in Sir2 homolog (SIRT1)-deficient mice. Proc Natl Acad Sci USA.

[19] Blander G, Guarente L (2004). The Sir2 family of protein deacetylases. Annu Rev Biochem.

[20] Denu JM (2003). Linking chromatin function with metabolic networks: Sir2 family of NAD(+)-dependent deacetylases. Trends Biochem Sci.

[21] Lin SJ, Kaeberlein M, Andalis AA, Sturtz LA, Defossez PA, Culotta VC, Fink GR, Guarente L (2002). Calorie restriction extends *Saccharomyces cerevisiae* lifespan by increasing respiration. Nature.

[22] Lin SJ, Ford E, Haigis M, Liszt G, Guarente L (2004). Calorie restriction extends yeast life span by lowering the level of NADH. Genes Dev.

[23] Belenky P, Racette FG, Bogan KL, McClure JL, Smith JS, Brenner C (2007). Nicotinamide riboside promotes Sir2 silencing and extends lifespan *via* Nrk and Urh1/Pnp1/Meu1 pathways to NAD+. Cell.

[24] Bordone L, Guarente L (2005). Calorie restriction, sirt1 and metabolism: understanding longevity. Nature Revs Mol Cell Biol.

[25] Medvedik O, Lamming DW, Kim KD, Sinclair DA (2007). MSN2 and MSN4 link calorie restriction and TOR to sirtuin-mediated lifespan extension in *Saccharomyces cerevisiae*. PLoS Biol.

[26] Hipkiss AR (2008). Energy metabolism, altered proteins, sirtuins and ageing: converging mechanisms?. Biogerontology.

[27] Dhindsa S, Tripathy D, Mohanty P, Ghanim H, Syed T, Aljada A, Dandona P (2004). Differential effects of glucose and alcohol on reactive oxygen species generation and intranuclear nuclear factor-κB in mononuclear cells. Metabolism.

[28] Salminen A, Ojala J, Huuskonen J, Kauppinen A, Suuronen T, Kaarniranta K (2008). Interaction of aging-associated signaling cascades: Inhibition of NF-κB signaling by longevity factors FoxOs and SIRT1. Cell Mol Life Sci.

[29] Raiser M, Wamelink MM, Kowald A, Gerisch B, Heeren G, Struys EA, Klipp E, Jakobs C, Breitenbach M, Lehrach H, Krobitsch S (2007). Dynamic rerouting of the carbohydrate flux is key to counteracting oxidative stress. J Biol.

[30] Di Loreto S, Zimmitti V, Sebastiani P, Cervelli C, Falone S, Amicarelli F (2008). Methylglyoxal causes strong weakening of detoxifying capacity and apoptotic cell death in rat hippocampal neurons. Int J Biochem Cell Biol.

[31] Thornalley PJ (2007). Endogenous alpha-oxoaldehydes and formation of protein and nucleotide advanced glycation endproducts in tissue damage. Novartis Found Symp.

[32] Hipkiss AR (2006). Caloric restriction and ageing—is glycolysis the problem?. Mech Ageing Dev.

[33] Cai W, He JC, Zhu L, Chen X, Wallenstein S, Striker GE, Vlassara H (2007). Reduced oxidant stress and extended lifespan in mice exposed to a low glycotoxin diet. Association with increased AGER1 expression. Am J Pathol.

[34] Uribarri J, Cai W, Peppa M, Goodman S, Ferrucci L, Striker G, Vlassara H (2007). Circulating glycotoxins and dietary advanced glycation endproducts: two links to inflammatory response, oxidative stress, and aging. J Gerontol A Biol Sci Med Sci.

[35] Lin SJ, Defossez PA, Guarente L (2000). Requirement of NAD and SIR2 for life-span extension by calorie restriction in Saccharomyces cerevisiae. Science.

[36] Utsugi T, Ohno T, Ohyama Y, Uchiyama T, Saito Y, Matsumura Y, Aizawa H, Itoh H, Kurabayashi M, Kawazu S, Tomono S, Oka Y, Suga T, Kuro-o M, Nabeshima Y, Nagai R (2000). Decreased insulin production and increased insulin sensitivity in the klotho mutant mouse, a novel animal model for human aging. Metabolism.

[37] McCay CM, Cromwell MF, Maynard LA (1935). The effect of retarded growth upon the length of life span and ultimate body size. J Nutr.

